# Kinetic Study of *In Vitro* Release of Neem from Chitosan Biopolymer and Assessment of Its Biological Effectiveness

**DOI:** 10.3390/polym17050702

**Published:** 2025-03-06

**Authors:** Yasodani Nishshanka, Charitha Thambiliyagodage, Madara Jayanetti

**Affiliations:** Faculty of Humanities and Sciences, Sri Lanka Institute of Information Technology, New Kandy Road, Malabe, Colombo 10115, Sri Lanka; y.a.nishshanka17@gmail.com (Y.N.); madara.ja@sliit.lk (M.J.)

**Keywords:** *Azadirachta indica*, chitosan, sustained drug release, antioxidant, anti-inflammatory, antibacterial

## Abstract

The study examined the sustained release of neem from the polymeric carrier system chitosan by varying the drug content, ionic strength of the release medium, and pH. Six different kinetic models, i.e., Korsmeyer–Peppas (KP), Peppas–Sahlin (PS), Higuchi, Hixson–Crowell, Zero order, and First order were used to investigate the drug release kinetics. Based on the R^2^ values, the KP and PS models were chosen from the examined models to study the drug release mechanism from the chitosan biopolymer. The values found for model parameters *n* and *m* in the KP and PS models differ noticeably, suggesting that Fickian diffusion and Case II relaxation are important components of the neem release mechanism from chitosan. At lower ionic strengths and lower pH values, neem is released from the composite mostly by Fickian diffusion. The diphenyl-2-picrylhydrazyl assay served to assess the composite’s antioxidant properties. The composite’s antioxidant properties ranged from 3.56 ± 1.89% at 10 μg/mL to 51.28 ± 1.14% at 70 μg/mL. The ability of the composite to inhibit the denaturation of egg albumin was also tested and it ranged from 59.68 ± 0.93% at 25 μg/mL to 187.63 ± 3.53% at 1600 μg/mL. The drug composite has exhibited antibacterial activity against *Klebsiella pneumoniae*, *Pseudomonas aeruginosa*, *Escherichia coli*, and *Staphylococcus aureus*, and proved to be highly effective against *P. aeruginosa* at lower concentrations and against *S. aureus* at higher concentrations. The resulting inhibition zones for *P. aeruginosa* at 5 and 10 mg/mL concentrations were 16.5 ± 2.25 mm, and 14.83 ± 0.6 mm, respectively, whereas for *S. aureus,* it was 16.67 ± 0.33 mm at 20 mg/mL. The neem–chitosan composite’s minimum inhibitory concentration/minimum bactericidal concentration ratio for *K. pneumoniae*, *P. aeruginosa*, and *S. aureus* was greater than 4, suggesting that they trigger bacteriostatic outcomes, whereas for *E. coli,* it was 4, which means that bactericidal effects were evident.

## 1. Introduction

Plants have historically been the primary source of medicine due to their production of minimal side effects, less toxicity, and affordability. Researchers aim to integrate alternative medicine with evidence-based medicine and better understand the metabolic processes and effects on the human body. For this reason, phytotherapy has garnered increasing attention in drug discovery studies [1,2]. *Azadirachta indica* is a tree native to Asia that has long been regarded as a universal remedy and is considered “nature’s drug store” [3]. The phytochemicals present in neem can be classified into two main groups: isoprenoids and non-isoprenoids. Isoprenoids encompass various compounds, including diterpenoids, triterpenoids, vilasinin types of compounds, protomeliacins, genin, azadirone, limonoids, and their derivatives, as well as C-secomeliacins like galanin, Nimbin, and azadirachtin. Non-isoprenoids comprise proteins, polysaccharides, sulfurous compounds, polyphenolics such as flavonoids and their glycosides, dihydrochalone, coumarin, tannins, and aliphatic compounds [4,5]. The most biologically active neem compound is azadirachtin, a blend of seven isomeric compounds identified as azadirachtin A to G. Among these, azadirachtin E has demonstrated superior effectiveness [6]. Neem leaves possess antifungal, antibacterial, antifertility, anti-inflammatory, antipyretic, anticancer, antigenotoxic, hepatoprotective, analgesic, antiulcerogenic, neuropharmacological, antihypertensive, immunostimulant, antioxidant, antihyperglycemic, anti-dermatophytic, and oro-dental protective properties [1,3,7,8].

Neem-derived bioactive compounds are compelling alternatives for treating various diseases, primarily due to their superior therapeutic efficacy, safety, lower toxicity, and affordability. Nevertheless, harnessing the therapeutic effects of *Azadirachta indica* relies significantly on its bioavailability within the human body. The bioactive compounds’ physicochemical properties and structural complexity significantly impact the challenges associated with bioavailability. They tend to change the structure in response to pH levels in the body, are not easily absorbed, and engage in unintended interactions with non-target substances [9,10]. Using polymeric delivery systems to transport bioactive compounds presents a promising approach to address this issue. This facilitates the absorption and distribution of drugs in the body, enhancing the efficacy and bioavailability of plant-derived compounds [11].

Medications introduced through the digestive and circulatory systems encounter obstacles. The body can reject drugs, while enzymes in the gastrointestinal tract and organs can biologically degrade specific components of the drugs. This results in insufficient delivery of the correct chemical composition to the diseased tissue at the necessary concentration [11]. In medical applications, drug delivery systems are crucial in achieving targeted delivery of pharmaceutical agents to specific locations, at required concentrations and timings. Controlled or sustained drug release systems have proven to be practical tools for treating various medical complications by enabling the delivery of drugs at a predetermined rate over a specified period. This approach helps to overcome challenges such as drug degradation and low bioavailability associated with traditional drug delivery methods [12,13,14]. This controlled delivery depends upon the size, shape, and internal structures of the used biopolymers [11].

Polymeric delivery systems such as liposomes, ethosomes, phytosomes, solid–lipid nanoparticles, emulsions, and microspheres have been employed for efficient drug delivery [9,11,15,16]. Various water- and lipid-soluble bioactive compounds extracted from plants have been encapsulated in liposomes. Bioactive compounds with medicinal properties, such as anthocyanin, betanin, carotenoids, curcumin, phytosterols, quercetin, etc., have been transported using different formulations of liposomes to improve their sustained release, bioaccessibility and bioavailability [17]. A study was conducted to increase the bioavailability of total flavonoids of *Dracocephalum moldavica*. In this study, they prepared the composite (TFDM-CPL) by encapsulating the extract in phospholipid liposomes, tested the drug release *in vitro* and conducted a drug pharmacokinetics study in Sprague-Dawley rats. The results showed an increased water solubility and bioavailability of the extract, while the composite showed excellent sustained release properties both *in vitro* and *in vivo* [18]. In another study, the hepatoprotective activity of *Blumea lacera* leaf extract was investigated. A liposomal encapsulation of the leaf extract was prepared to improve the drug efficiency and overcome the barriers of poor solubility, permeability, and bioavailability. The Blumea lacera leaf extract delivered using the liposomal nano drug delivery system showed better efficacy than the leaf extract [19]. Ethosomes and transethosomes have been widely used in drug delivery, especially for transdermal delivery of drugs. These polymeric delivery systems are very flexible and can maintain the stability of the chemical compounds and control drug release. Several studies report the delivery of plant extracts containing mangiferin, quercetin, curcumin, catechin, rutin, etc., using ethosomes and transethosomes as the nanocarriers [20]. Research was carried out to develop an autosomal gel with *Brassica oleraceae* L. to enhance the extract’s drug release properties. This has shown improved permeability and an efficient sustained release of the plant extract encapsulated ethosomal gel, potentially used in both cosmeceutical and pharmaceutical fields [21]. Phytosomes are a rapidly evolving group of carriers that can be used for oral, topical, and transdermal delivery of drugs. They have a high encapsulation efficiency and the ability to release the compounds slowly. Bioactive compounds like curcumin, quercetin, etc., and plant extracts obtained from *Ocimum sanctum*, *Melilotus officinalis* (L.), *Centella asiatica,* etc., have been delivered in phytosomes to different parts of the human body in previous studies [22]. Solid–lipid nanoparticles have become a popular nanocarrier due to their controlled and targeted release efficiency, increased surface area and higher encapsulation efficiency. The use of solid–lipid nanoparticles to carry bioactive compounds like Epigallocatechin-3-gallate, curcumin, quercetin, resveratrol, etc., and plant extracts such as pomegranate extract, grape seed and skin extracts, etc., has been reported in the literature [23].

Biopolymers derived from natural materials have gained increased interest in different fields because of their biological origin, non-toxicity, and biodegradability. These can be developed from living organisms or renewable sources. Cellulose, starch, chitin/chitosan, and alginates are commonly known biopolymers [16,24]. Among those, chitosan is a versatile polymer and is important due to its macromolecular structure and physicochemical properties. It has a range of biological and pharmacological properties as well. It is a linear polymer composed of d-glucosamine and N-acetyl-d-glucosamine. Chitosan is a biodegradable and biocompatible polymer incorporating key properties, including antioxidant, anti-inflammatory, antihypertensive, anticoagulant, antitumoral, antidiabetic, antimicrobial, and hypocholesterolemic characteristics [25,26]. Chitosan holds promise as a material for regulated drug release, making it possible to design formulations suitable for administering drugs through either oral or intravenous routes [27]. Chitosan has been employed to deliver many drugs, including curcumin [28], quercetin [29], gingerol [30], rosmarinic acid [31], and limonene [32]. Chitosan was selected as the drug carrier in the present study, as it has shown promising sustained release properties in previous research, particularly for plant extracted bioactive compounds. Furthermore, the ability to synthesize the polymer cost-effectively from a waste material that is of biological origin, the convenience of the synthesis procedure, the eco-friendly and user-friendly nature, and its status as a polymer with pharmacological properties that are compatible with the properties of the studied plant extract encouraged the selection of chitosan.

Evidence from previous research supports the use of various polymers for transporting bioactive compounds of neem and the use of biopolymer chitosan for delivering certain drugs. Notably, a combination of neem leaf extract and alginate chitosan nanofibers has demonstrated successful results as a drug delivery system with antioxidant and antifungal properties [9]. In another study, a novel drug delivery system involving neem ethanolic leaf extract with nano-chitosan was tested against the pancreatic histology of a species of white male rats [11]. Additionally, a study was conducted to design, develop, and characterize liposomal neem gel by combining methanolic neem extract with liposomes using the thin-film hydration method. This study exhibited excellent *in vitro* drug diffusion and skin retention rates of the novel drug [30]. Furthermore, an attempt to prepare PCL/PVA/chitosan containing neem extract-loaded phenylalanine nanotubes has been successfully utilized as a delivery system for oral and dental therapeutic applications [33]. N-trimethyl chitosan chloride has been investigated and demonstrated potential in hydrogel formulations for nasal drug delivery. Chitosan has also been explored as an injectable vehicle for drug delivery in the presence of sodium bicarbonate (NaHCO_3_), resulting in a porous hydrogel with variable drug release behaviors contingent on the NaHCO_3_ concentration. Additionally, a study has been conducted employing chitosan oligomer zidovudine composites as a drug delivery system for the *in vitro* release of the drug zidovudine. This study, including a pharmacokinetics analysis using mice plasma and renal homogenate, has indicated a prolonged retention time for the chitosan oligomer–zidovudine conjugate compared to zidovudine alone. This suggests its potential for renal-targeting drug delivery [34].

This study determined the enhanced bioavailability of *Azadirachta indica* leaf extract by incorporating it into the natural biopolymer chitosan, creating a composite that acts as a sustained drug delivery system. The ethanolic crude extract of the neem leaves was obtained, chitosan was prepared from shrimp shell waste material, and the neem–chitosan drug composite was developed. A phytochemical analysis was conducted to verify the presence of bioactive compounds in the leaf crude extract, and an SEM and XRD analysis was conducted to characterize the composite. The *in vitro* release of neem extract from chitosan under different conditions was investigated. Further, the proposed drug’s antioxidant properties, potential to inhibit the denaturation of egg albumin, and antibacterial properties were studied. While previous studies have coupled different neem extracts with various types of polymers and chitosan with a wide variety of plant extracts to design composites for use in various industries, the specific exploration of the above properties of neem leaf extract coupled with chitosan as a drug delivery system in the pharmaceutical field has not yet been reported in the literature.

## 2. Materials and Methods

### 2.1. Preparation of Neem Leaf Crude Extract

Fresh and healthy neem leaves were collected and washed under running tap water for approximately 5 min. The washed leaves were left to dry at room temperature for one day and subjected to oven-drying at 40 °C for 22 h. Once dried, the leaves were finely chopped and ground into a powdered form using a dry and clean blender. A double organic extraction was conducted using ethanol. First, 5 g of neem leaf powder and 25 mL of 80% ethanol were introduced into two 50 mL polypropylene centrifuge tubes. The tubes were then securely sealed and placed in an ultrasonic bath at 40 °C for 3 h. Subsequently, the mixture was centrifuged at 3000× *g* rpm for 4 min. The obtained liquid extract was transferred to a 100 mL volumetric flask, covered with aluminum foil, and stored in the refrigerator at 4 °C. The residual leaf matter from the first extraction was subjected to the second extraction, which was conducted using the same procedure. Then, the second extract was mixed and stored with the first extract. Lastly, the liquid extract was transferred to an evaporating dish, covered with aluminum foil, and placed on a hot plate at 40 °C for 2–3 days to facilitate the evaporation of the ethanol solvent. The extracted powder was stored in the refrigerator at 4 °C for further utilization.

### 2.2. Synthesis of Chitosan Biopolymer from Chitin

Shrimp shell waste was collected from two species, *Penaeus monodon* (Giant Tiger Prawn) and *Penaeus vannamei* (Whiteleg Shrimp). They were thoroughly cleaned using tap water, distilled water, and 70% Isopropyl Alcohol (IPA) and were oven-dried at 80 °C for 3 to 4 h. Then, the dried shells were ground into a fine powder and subjected to demineralization, deproteinization, and deacetylation. Shrimp shell powder was treated with 10% HCl and then subjected to continuous stirring at 500 rpm at room temperature for 24 h to facilitate demineralization. Then, the 10% HCl solution was decanted, and the demineralized shrimp shell powder was thoroughly rinsed with distilled water until a neutral pH was achieved. Following this, the sample was treated with a 3% NaOH solution accompanied by continuous stirring at 450 rpm at room temperature for 5 h to facilitate the deproteinization. After the deproteinization process, the 3% NaOH solution was removed, and the sample was treated with a 50% NaOH solution through reflux condensation to facilitate deacetylation. The sample temperature was maintained at 80 °C and continued for 2 h. Finally, the 50% NaOH solution was decanted, and the prepared chitosan was thoroughly washed with distilled water until it reached a neutral pH and oven-dried at 80 °C [35,36].

### 2.3. Preparation of Neem–Chitosan Composite

The prepared chitosan powder (1 g) and neem ethanolic extract (100 mL) were mixed and heated on a magnetic stirrer at 40 °C and stirred at 100 rpm until the ethanol solvent was completely evaporated. The resulting composite was oven-dried at 40 °C for 2–3 h.

### 2.4. Phytochemical Analysis of Neem Leaf Extract

Tests for proteins, carbohydrates, phenols and tannins, flavonoids, saponins, glycosides, steroids, terpenoids, and alkaloids were conducted in the phytochemical analysis. A 4 mg/mL solution of neem leaf extract was prepared using methanol. A total of 250 μL of this solution was used for each test. The experiments were performed in triplicate.

#### 2.4.1. Test for Proteins

##### Ninhydrin Test

The neem leaf extract was boiled with 250 μL of 0.2% Ninhydrin solution. Violet color indicates the presence of proteins.

#### 2.4.2. Test for Carbohydrates

##### Fehling’s Test

Fehling A and Fehling B reagents were mixed in equal volumes and 250 μL of it was added to the neem leaf extract. The resulting mixture was gently boiled. Brick red precipitation occurs in the presence of carbohydrates.

##### Benedict’s Test

The neem leaf extract was mixed with 250 μL of Benedict’s reagent and the solution was boiled. Reddish brown precipitation occurs in the presence of carbohydrates.

##### Iodine Test

The neem leaf extract was mixed with 250 μL of iodine solution. Dark blue/purple color in the solution indicates the presence of carbohydrates.

#### 2.4.3. Test for Phenols and Tannins

The neem leaf extract was mixed with 250 μL of 2% FeCl_3_ solution. Blue green/black color indicates the presence of phenols and tannins.

#### 2.4.4. Test for Flavonoids

##### Alkaline Reagent Test

The neem leaf extract was mixed with 250 μL of 2% NaOH solution. Then, a few drops of dilute H_2_SO_4_ were added to the resulting solution. The intense yellow color that appears after the addition of NaOH becomes colorless after dilute H_2_SO_4_ is added, if flavonoids are present.

#### 2.4.5. Test for Saponins

The neem leaf extract was mixed with 250 μL of distilled water and shaken vigorously. A stable form indicates the presence of saponins.

#### 2.4.6. Test for Glycosides

##### Salkowski’s Test

The neem leaf extract was mixed with 250 μL of chloroform. Then, 250 μL of concentrated H_2_SO_4_ was added and shaken gently. Reddish brown color indicates the presence of glycosides.

##### Keller–Kilani Test

The neem leaf extract was mixed with 250 μL of glacial acetic acid and a few drops of 2% FeCl_3_ solution. This was mixed with 250 μL of concentrated H_2_SO_4_ added to another test tube. A brown ring at the interphase indicates the presence of glycosides.

#### 2.4.7. Test for Steroids

The neem leaf extract was mixed with 250 μL of chloroform, and concentrated H_2_SO_4_ was added from a side of the test tube. A red color is produced in the lower chloroform layer if steroids are present.

#### 2.4.8. Test for Terpenoids

The neem leaf extract was dissolved in 250 μL of chloroform and evaporated until dry. Then, 250 μL of concentrated H_2_SO_4_ was added and heated for 2 min. A grayish color appears in the presence of terpenoids.

#### 2.4.9. Test for Alkaloids

The neem leaf extract was mixed with 250 μL of 1% HCl and heated gently. This was followed by the addition of Mayer’s and Wagner’s reagents. If the resulting precipitation is turbid, it indicates the presence of alkaloids.

### 2.5. Determination of the Antioxidant Activity

The antioxidant activity of neem leaf extract and composite was determined by evaluating their ability to scavenge the stable free radical DPPH (1, 1-diphenyl-2-picrylhydrazyl). Seven sample concentrations ranging from 0.01 mg/mL to 0.07 mg/mL were prepared. All solutions were prepared using methanol as the solvent. 5 mL of each prepared sample was mixed with 0.5 mL of 1 mM DPPH solution in methanol. The control sample was prepared without the analyte. Following preparation, the samples were incubated in darkness at room temperature for 30 min. Absorbance readings were then taken at 517 nm. The entire experiment was executed in triplicate. Ascorbic acid functioned as the positive control in the assay.

The ability of the various neem leaf extract and composite concentrations to scavenge the DPPH radical was calculated using the following equation [37].

(1) $$\%\;\mathrm{Radical}\;\mathrm{Scavenging}\;\mathrm{Activity}=\frac{Abs\;of\;control-\mathrm{Abs}\;\mathrm{of}\;\mathrm{sample}}{Abs\;of\;control}\;\text{×}100$$

### 2.6. Determination of the Ability to Inhibit the Denaturation of Egg Albumin

Seven sample concentrations ranging from 0.025 mg/mL to 1.6 mg/mL were prepared. All solutions were prepared using methanol as the solvent. A total of 2 mL of each prepared sample was mixed with 0.2 mL of 1% egg albumin solution and 2.8 mL of phosphate-buffered saline (pH 6.4) to prepare the reaction mixture. The control sample was prepared without the analyte. Following preparation, the samples were incubated at 37 ± 2 °C for 30 min and subsequently heated in a water bath at 70 ± 2 °C for 15 min. Then, the absorbance readings were taken at 660 nm. The entire experiment was performed in triplicate. Diclofenac sodium was used as the positive control in the assay.

The ability of the various neem leaf extract and composite concentrations to inhibit protein denaturation was calculated using the following equation [38].

(2) $$\%\;\mathrm{Inhibition}=\frac{Abs\;of\;sample-\mathrm{Abs}\;\mathrm{of}\;\mathrm{control}}{Abs\;of\;control}\;\text{×}100$$

### 2.7. Determination of the Antibacterial Activity

The antibacterial activity of the neem leaf extract, chitosan, and composite was assessed using the agar well diffusion method. From each of the three samples, 5 mg/mL, 10 mg/mL, and 20 mg/mL concentrations were prepared in dimethyl sulfoxide (DMSO) and placed in an ultrasonic bath (Sonicator, PCL Analytics, Maharashtra, India) for 1 h at 40 °C. The study used three Gram-negative bacterial strains: *Escherichia coli, Klebsiella pneumoniae,* and *Pseudomonas aeruginosa*, and one Gram-positive bacterial strain, *Staphylococcus aureus*. Bacterial suspensions were prepared in Luria–Bertani (LB) medium and incubated for 24 h at 37 °C. Culture plates were prepared using Mueller–Hinton agar (MHA) growth medium. The 24 h aged bacterial cultures were adjusted for broth dilution to obtain 5 × 10^5^ CFU/mL with a 0.5 McFarland turbidity standard as the visual indication and a spectrophotometer [39]. The bacterial inoculums were transferred onto Mueller–Hinton agar plates using a sterile cotton swab. Wells were then prepared and the prepared samples were introduced. All the culture plates were incubated at 37 °C for 6 to 18 h. Following the incubation period, the zones of inhibition were measured. The same procedure was employed for the control plates. Amoxicillin (positive control), DMSO (negative control), ethanol, and distilled water were used as control samples. The entire experiment was performed in triplicate.

#### 2.7.1. Determination of the Minimum Inhibitory Concentration

The Broth dilution method determined the minimum inhibitory concentration (MIC). Bacterial suspensions were prepared and adjusted as in the prior experiment. From each of the three samples, 40 mg/mL concentration was prepared in DMSO followed by placing it in an ultrasonic bath (Sonicator) for 1 h at 40 °C. A series of two-fold serial dilutions were performed, resulting in six concentrations ranging from 40 mg/mL to 1.25 mg/mL of each of the three samples. To each test tube containing a distinct concentration of the sample, the broth culture was added in a 1:1 ratio. The bacterial culture was introduced solely as the control for one test tube. This procedure was carried out for all four bacterial strains. Subsequently, the test tubes were incubated at 37 °C for 24 h. The turbidity of the samples indicated the presence of bacterial growth. The MIC was determined to be the minimum concentration of the samples which led to no visible bacterial growth.

#### 2.7.2. Determination of the Minimum Bactericidal Concentration

Culture plates were prepared using Mueller–Hinton agar (MHA) growth medium. To determine the minimum bactericidal concentration (MBC) of all three samples, 0.2 mL of the bacterial suspension from the test tubes where MIC determination was conducted was transferred onto agar plates. The bacterial suspension was evenly spread across the agar surface using a sterile glass spreader. Subsequently, the Petri plates were incubated at 37 °C for 24 h. After the completion of the incubation period, the colonies were counted and recorded. The concentration at which no colony formation was observed was recorded as the MBC.

### 2.8. Drug Release Kinetics Study

#### 2.8.1. *In Vitro* Drug Release Analysis

The release kinetics of the neem–chitosan composite were studied using the spectrophotometric method. The instrument used to measure the drug release was the UV-Vis Plus Spectrophotometer, Shimadzu 1900, Kyoto, Japan. The release of neem from chitosan was evaluated *in vitro* under various pH conditions and ionic mediums over 15 min intervals. A total of 5 mg of the composite was introduced into a cuvette containing the respective medium, and the maximum and minimum absorbance values were recorded. The pH mediums employed were pH 1, pH 2.5, pH 4, pH 5.5, pH 7, pH 7.4, pH 8.5, and pH 10. Ionic mediums of 0.1 M, 0.2 M, 0.3 M, 0.4 M, and 0.5 M NaCl were utilized. A curve was plotted by correlating the differences in absorbance against time, and data were collected until the curve reached a plateau. The pH and ionic mediums showing the most effective drug release were combined (pH 7.4 and 0.4 M NaCl), and the drug release in the combined medium was analyzed using different weights of the composite (2.5 mg, 5 mg, 7.5 mg, and 10 mg). All experiments were conducted in triplicate.

#### 2.8.2. Drug Release Kinetic Models

Six models were employed to examine the release kinetics, including the Korsmeyer–Peppas, Peppas–Sahlin, Higuchi, zero-order, first-order, and Hixson–Crowell models. The absorbance readings were converted to percentages of cumulative drug release (CDR%) before the drug release kinetics study [40].

(3) $$CDR\left(\%\right)=\frac{Absorbance\;difference}{Active\;neem\;weight\;in\;composite}\times 100$$

The Korsmeyer–Peppas Model is as follows:

(4) $$f_{1}=\frac{M_{i}}{M_{\infty }}=Kt^{n}$$

where $f_{1}$ is the amount of drug released, $M_{\infty }$ denotes the amount of drug at the equilibrium state, $M_{i}$ stands for the amount of drug released over time t, K represents the release velocity constant, and n is the exponent of release as the function of time t.

The Peppas–Sahlin model is as follows:

(5) $$\frac{M_{t}}{M_{\infty }}=K_{d}t^{m\;}+K_{r}t^{2m\;}$$

where $K_{d}$, $K_{r}$, and m are constants. Considering the right side of the equation, the first term represents the Fickian diffusional contribution, F, whereas the second term represents the Case II relaxational contribution, R.

The Higuchi Model is as follows:

(6) $$f_{1}=Q=K_{H}\sqrt{t}$$

where $K_{H}\;$is the release constant of the Higuchi model.

The Zero-Order Model is as follows:

(7) $$f_{i\;}=K_{0}t$$

where $K_{0}$ is a constant of the apparent velocity of dissolution.

The First-Order Model is as follows:

(8) $${\log Q}_{1}={\log Q}_{0}+\frac{k_{1}t}{2.303}$$

where $Q_{1}\;$is the amount of active agent released at time t, $Q_{0}\;$denotes the initial amount of drug dissolved, and $k_{1}$ stands for the first-order constant.

The Hixson–Crowell Model is as follows:

(9) $$\sqrt[{3}]{W_{0\;}}=\sqrt[{3}]{W_{i}}+K_{HC}t\;$$

where $W_{0\;}$ is the initial amount of the drug in the system; $W_{i}$ is the amount remaining in the system at time t; and $K_{HC}$ is the constant of incorporation, which relates surface and volume.

#### 2.8.3. Statistical Analysis of Drug Release Studies

Standard deviation (SD) values and the correlation coefficient (R^2^) values were used to select the kinetic model that fits the data precisely. This was used to study the drug release mechanism. Statistical analysis using one-way analysis of variance (ANOVA) was used to assess the differences between the obtained release profiles at different pH and ionic mediums. A statistically significant difference was indicated when *p* < 0.05. ANOVA was conducted using the SPSS Statistics software 25.0 (IBM, SPSS Inc., Chicago, IL, USA).

### 2.9. Characterization

The synthesized chitosan and composite were characterized using ZEISS EVO 18 RESEARCH field-emission SEM, Jena, Germany, apparatus operating at an acceleration voltage of 0.2 to 30 kV and a magnification of <5–1,000,000× to study the surface morphology of chitosan and composite. The crystallography of the neem powder, synthesized chitosan, and the composite was determined using the Rigaku Ultima IV apparatus, Tokyo, Japan. It was operated with Cu Kα (λ = 0.154) radiation, varying the 2θ with a scanning range from 10° to 80°, at a scan speed of 2° per minute. The d spacing values were calculated utilizing Bragg’s law, as shown in Equation (10). The crystallite size of the materials was calculated using the Scherrer equation, as shown in Equation (11), using the peaks with the highest intensity.

Bragg’s Law:

(10) nλ=2d sinθ

Scherrer Equation:

(11) $$L=\frac{K\lambda }{\beta \;cos\theta }$$

*n*: constant, *λ*: wavelength of the x-ray, *d*: distance between lattice planes, *θ*: diffraction angle, *L*: crystallite size, *K*: Scherer’s constant, *β*: half maximum of the peak (radians)

## 3. Results and Discussion

### 3.1. Phytochemical Analysis

Phytochemical analysis was conducted to detect the presence of major classes of bioactive compounds and validate the results obtained in assessing antioxidant properties, inhibition of egg albumin denaturation, and antibacterial properties. Results showed (Table 1) the presence of phenols and tannins, flavonoids, glycosides, steroids, and terpenoids in the obtained ethanolic extract. Literature evidence provides the presence of proteins, carbohydrates, saponins, and alkaloids in different extracts of neem leaves, which were not detected in the present study [41]. Even though more than 300 bioactive compounds have been isolated from neem plants, individual trees have shown variations in their chemical composition. This may be due to the genetic influence and the environmental factors that affect the individual plants [42]. Another reason for the diversity in phytochemicals detected is the solvent used for the extraction process. In the present study, crude extract was obtained through an ethanolic extraction to increase yield, as documented in prior research. However, other studies conducted using water, dichloromethane, methanol, chloroform, acetone, hexane, etc., as solvents have shown variations in the isolated phytochemicals [41]. Apart from this, extraction conditions like type and concentration of the solvent, temperature and pressure used for the extraction, extraction time, pH of the medium, and even the particle size of the used plant material and plant material-to-solvent ratio play an important role in the extraction efficiency of different plant bioactive compounds [43].

The presence of terpenoids may provide insight into the presence of limonoids in the neem leaf extract. The limonoids obtained from the neem plant include azadirachtin, nimbin, and salannin, the main bioactive compounds in neem. Other than terpenoids, compounds like phenols, tannins, and flavonoids possess a wide array of medicinal attributes, including antioxidant and antimicrobial properties and the ability to inhibit the denaturation of egg albumin, which were important in this study [1,44,45,46,47].

### 3.2. Scanning Electron Microscopy (SEM) Analysis

**Figure 1:**
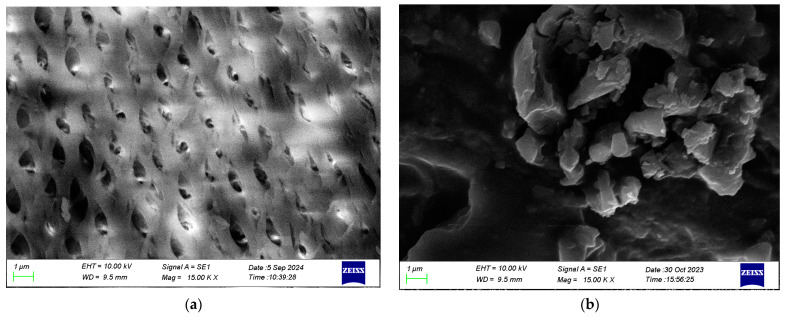
SEM image of (**a**) chitosan, (**b**) neem–chitosan composite.

The SEM analysis was undertaken to visualize the surface morphology of chitosan biopolymer and neem–chitosan composite. The analysis has revealed a flat, smooth, and uniform surface on chitosan. A well-established oval-shaped macropore structure has been organized on the chitosan surface with the removal of proteins present in chitin, as shown in Figure 1a. The neem extract has been crystallized on chitosan’s porous structure; hence, the well-established macropores are being disturbed, as shown in Figure 1b. Both micrographs are at a magnification of 15.00 KX and a scale of 1 µm.

### 3.3. X-Ray Diffractometry (XRD) Analysis

**Figure 2:**
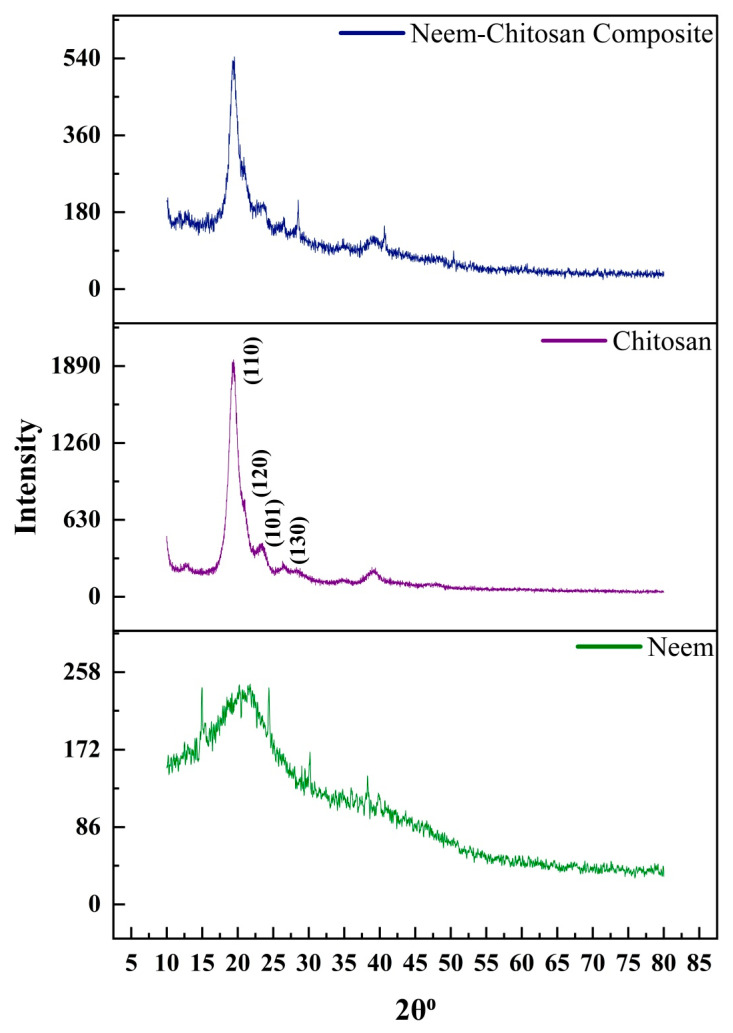
The XRD patterns of neem, chitosan and neem–chitosan composite.

The XRD analysis investigated the crystallographic orientation of neem leaf powder, chitosan biopolymer, and neem–chitosan composite (Figure 2). The XRD pattern of the neem leaf powder shows four major peaks at 14.92°, 20.36°, 24.44°, and 30.12°, attributed to the (101), (200), (211), (202) crystalline planes of chitosan, respectively. The d spacing values for 14.92°, 20.36°, 24.44°, 30.12°, 38.50° and 40.90° are 0.5931, 0.4357, 0.3638, 0.2963, 0.2336, and 0.2204 nm, respectively. The chitosan XRD pattern shows a significant peak at 19.36°, which is attributed to the (110) crystalline plane. The shoulder peak at 21.02° is attributed to the (120) crystalline plane. Peaks present at 23.38° and 26.40° are attributed to, respectively, the (101) and (130) crystalline planes [48]. The d spacing values calculated for the peaks at 19.36°, 21.02°, 23.38°, 26.40° and 39.14° are 0.4579, 0.4221, 0.3800, 0.3372, and 0.2299 nm, respectively. The XRD pattern of the neem–chitosan composite shows peaks at 19.48°, 20.86°, 23.64°, and 26.48°, confirming the crystallization of neem extract on chitosan. All the crystallographic parameters calculated are tabulated in Table 2. Crystallization of neem on chitosan increased the crystallite size and the number of planes. The crystallographic parameters of chitosan and the composite are very close. This is because the content of neem crystallized on chitosan structure is comparatively lower. During the washing, all the loosely bound neem was removed, and only the neem which formed covalent and electrostatic interactions with chitosan and the neem which crystalized in the pore network of chitosan remained.

### 3.4. Determination of the Antioxidant Activity

**Figure 3:**
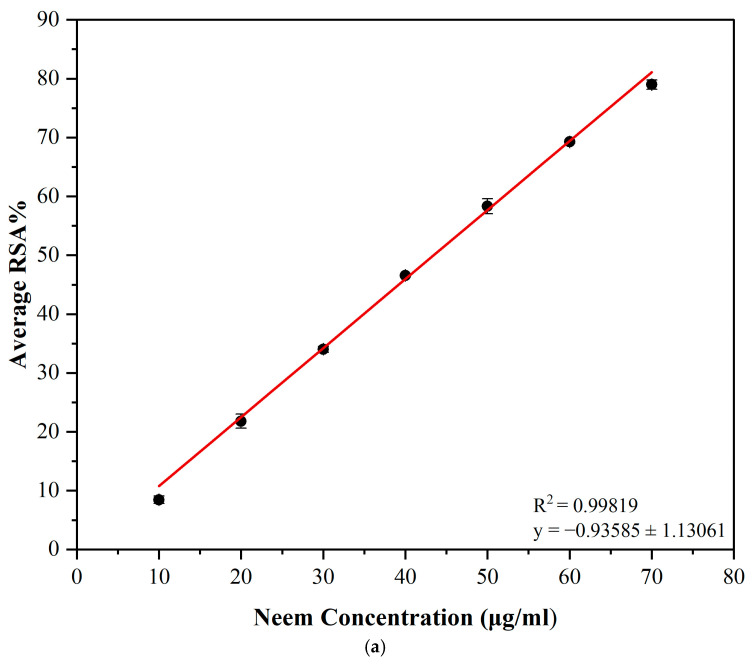

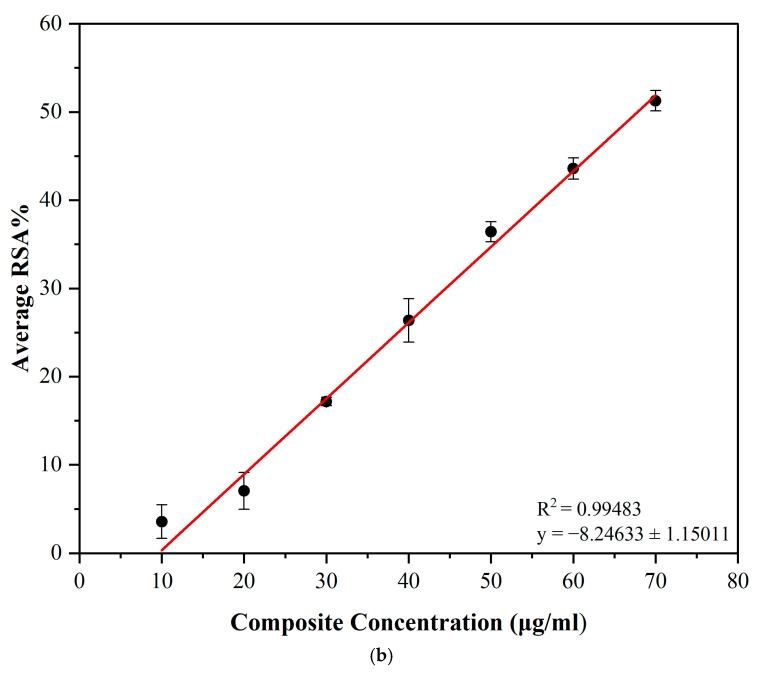
DPPH antioxidant activity of (**a**) neem extract and (**b**) neem–chitosan composite.

The ability of the neem leaf extract and the composite to scavenge DPPH was evaluated in the current study and their antioxidant activity was demonstrated. The variation of antioxidant activity of neem leaf extract and composite with increasing concentrations is shown in Figure 3a and Figure 3b, respectively. Both samples showed a linear increase in the antioxidant activity with increasing concentrations. The scavenging activity of the neem leaf extract ranged from 8.47 ± 0.64% at 10 μg/mL to 79.00 ± 0.79% at 70 μg/mL with an IC50 value of 43.45 μg/mL. Conversely, in the neem–chitosan composite, it ranged from 3.56 ± 1.89% at 10 μg/mL to 51.28 ± 1.14% at 70 μg/mL with an IC50 value of 67.80 μg/mL.

Neem leaf extract and the composite revealed higher scavenging activity in the DPPH assay. This specific ability should most likely be due to different phytochemicals showing superior antioxidant activity [1]. Neem leaves contain a large amount of bioactive compounds that provide radical scavenging activity. Specifically, limonoids, flavonoids and phenols are found to have a defensive function [45]. Flavonoids and phenols were identified as being present in the phytochemical analysis that was performed. Using ethanol to extract the crude extract from the leaves likely contributed to enhanced antioxidant activity, owing to its polar nature [49]. Chitosan can also chelate metal ions and attack free radicals.

However, the antioxidant activity of chitosan decreases with increasing molecular weight due to its poor solubility [35]. Chitosan also has a very low solubility in neutral solvents like methanol [50]. Since high-molecular-weight chitosan was produced, because the raw material used for the synthesis was shrimp shells and the DPPH assay was performed using methanol as the solvent, the composite’s solubility in the solvent was low. Therefore, the antioxidant activity of the neem–chitosan composite may have resulted from the combination of the pure neem leaf extract bound to chitosan and the small amount of chitosan dissolved in the medium. This resulted in a comparatively minimal amount of antioxidant activity in the composite compared to the pure neem extract. However, as the concentration of neem bound to chitosan is much lower in a given weight than that of the pure neem extract, the antioxidant activity of the composite can be considered significant. Table 3 shows the related studies conducted to test the antioxidant activity using DPPH assay.

### 3.5. Determination of the Ability to Inhibit the Denaturation of Egg Albumin

**Figure 4:**
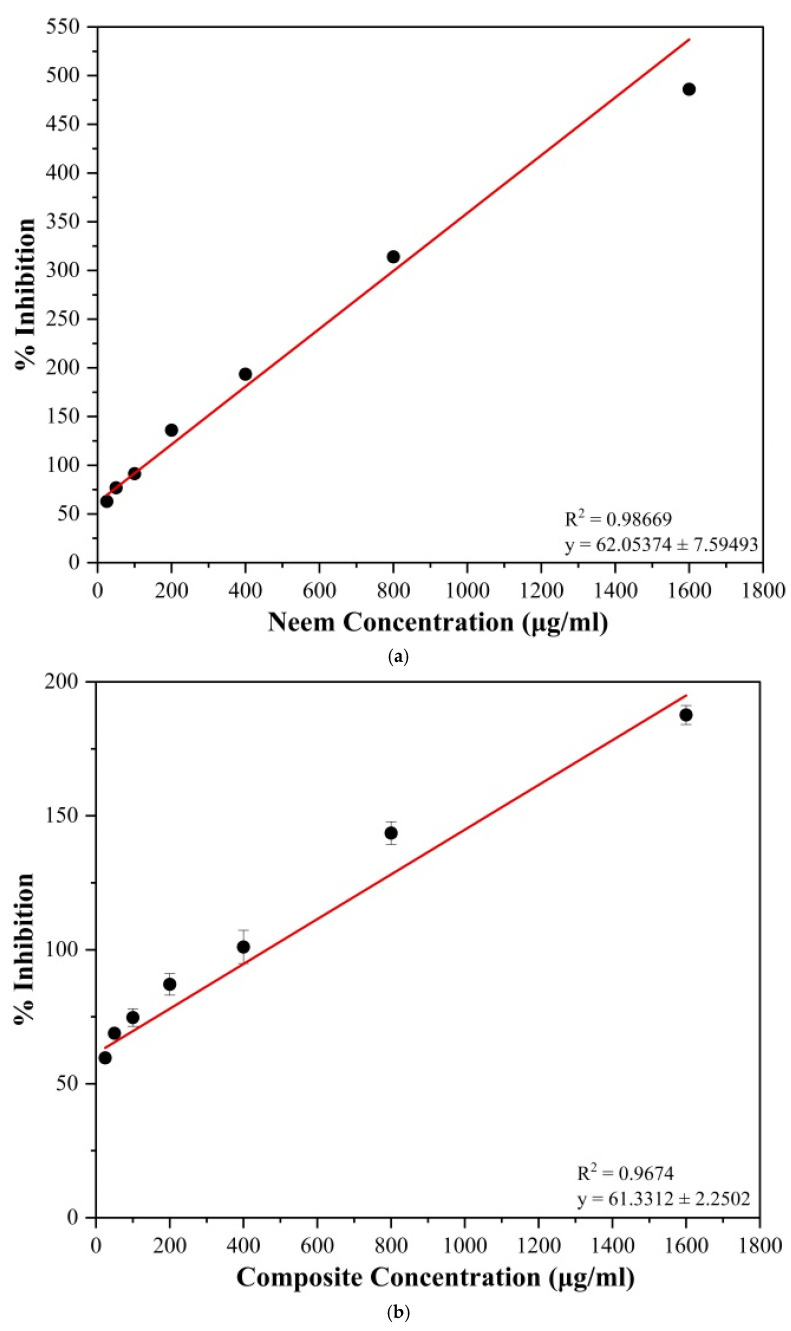
Ability to inhibit the denaturation of egg albumin of (**a**) neem extract and (**b**) neem–chitosan composite.

The ability of the neem leaf extract and composite to inhibit denaturation of the egg albumin protein was evaluated in the current study. The inhibition of protein denaturation by the neem leaf extract ranged from 62.90 ± 0.93% at 25 μg/mL to 486.02 ± 3.53% at 1600 μg/mL. In contrast, in the neem–chitosan composite, it ranged from 59.68 ± 0.93% at 25 μg/mL to 187.63 ± 3.53% at 1600 μg/mL. The variation of inhibition of neem leaf extract and neem–chitosan composite with increasing concentrations is shown in Figure 4a,b. Both samples showed a linear increase in the inhibition of protein denaturation with increasing concentrations.

Pure neem leaf extract showed higher inhibition against protein denaturation compared to the neem–chitosan composite. This ability is most likely due to different phytochemicals, especially limonoids and flavonoids, that have indicated superior inhibition [1]. The activity of the neem–chitosan composite is lower than that of pure neem extract, and its similarity resulted in antioxidant activity. This was due to, firstly, the small amount of neem active compounds present in the composite in a given weight, and secondly, the low solubility of the high-molecular-weight chitosan in methanol, as described in detail above. Table 4 exhibits related studies conducted to test the inhibition of denaturation of egg albumin.

### 3.6. Determination of the Antibacterial Activity

**Figure 5:**
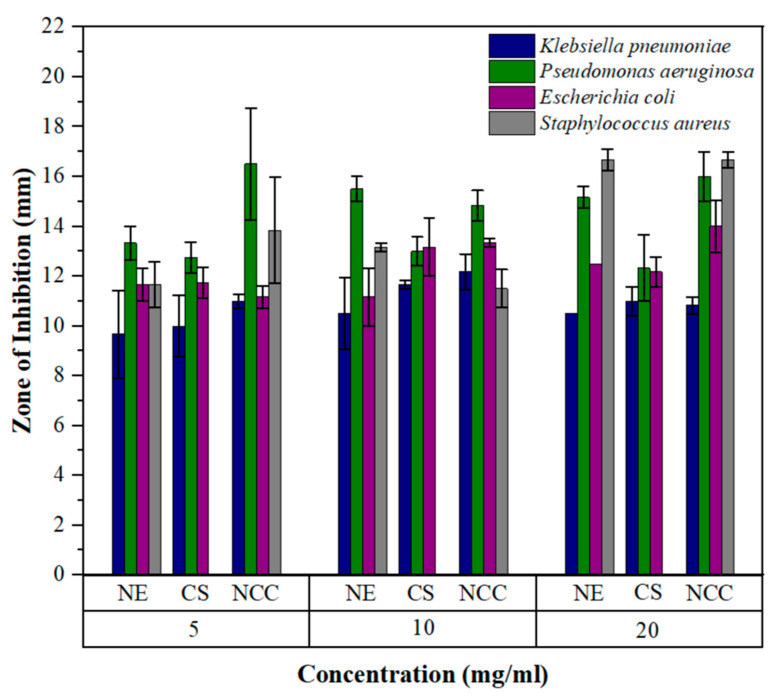
Antibacterial activity of neem (NE), chitosan (CS), neem–chitosan composite (NCC).

The antibacterial activity of neem (NE), chitosan (CS) and the neem–chitosan composite (NCC) was tested against *Escherichia coli*, *Staphylococcus aureus*, *Klebsiella pneumoniae* and *Pseudomonas aeruginosa* using three different concentrations, namely 5, 10 and 20 mg/mL. The results are illustrated in Figure 5.

The results of the antibacterial study revealed varying degrees of effectiveness of the tested samples in different concentrations against the growth of the tested bacterial strains. Neem leaf extract, chitosan, and neem–chitosan composite showed substantial antibacterial activity against all four bacterial strains except chitosan, which was not effective against *Staphylococcus aureus*.

The neem leaf extract exhibited a concentration-dependent antibacterial effect. The maximum inhibition of *Escherichia coli* and *Staphylococcus aureus* was observed at 20 mg/mL, with inhibition zones measuring 12.5 ± 0 mm and 16.67 ± 0.44 mm, respectively. Meanwhile, inhibition was similar for *Klebsiella pneumoniae* and *Pseudomonas aeruginosa* at 10 mg/mL and 20 mg/mL. The inhibition zones for 10 mg/mL were 10.50 ± 1.44 mm and 15.50 ± 0.50 mm, and for 20 mg/mL, they were 10.50 ± 0 mm and 15.17 ± 0.44 mm for *Klebsiella pneumoniae* and *Pseudomonas aeruginosa*, respectively. Notably, *Pseudomonas aeruginosa* demonstrated the highest susceptibility to neem leaf extract at 5 and 10 mg/mL. The lowest susceptibility was in *Klebsiella pneumoniae* for all three concentrations.

Even though the precise mechanism underlying the antibacterial activity of neem leaf extract has not been fully elucidated, it can be inferred that the presence of specific bioactive compounds, namely nimbidin, nimbin, nimbolide, gedunin, azadirachtin, mahmoodin, margolone, and cyclic trisulphide, is responsible for this effect [2,62].

Certain compounds, particularly flavonoids, exhibit the ability to, firstly, impact bacterial cell membranes and cell walls detrimentally; secondly, inhibit the synthesis of nucleic acids and proteins (including the inhibition of DNA topoisomerase I, II, and IV); thirdly, impede energy production (via inhibition of NADH cytochrome c reductase, succinate dehydrogenase, and malate dehydrogenase); and fourthly, hinder efflux pumps. Additionally, these compounds can elevate osmotic pressure within bacterial cells [44,63]. The extract diffused well through the agar medium, which generated good results in all the scenarios.

**Figure 6:**
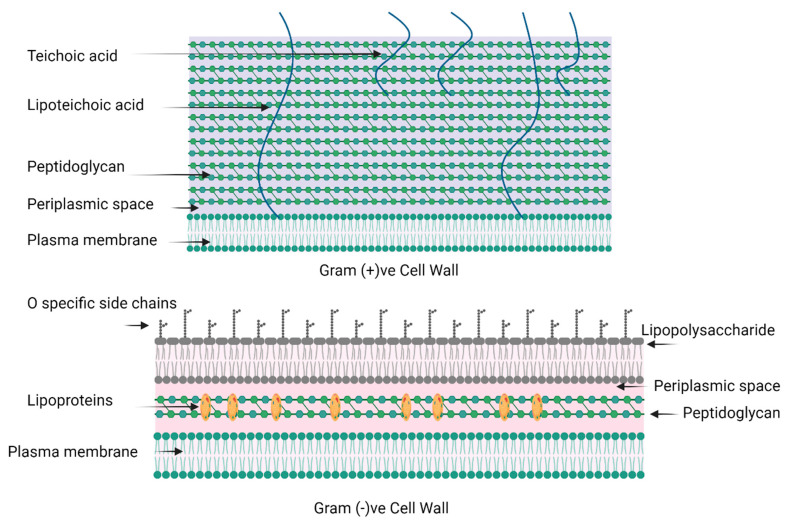
Schematic illustration of the cell walls of Gram-positive and Gram-negative bacteria.

Chitosan demonstrated the highest antibacterial effect at 10 mg/mL concentration across all tested strains except for *Staphylococcus aureus*. The inhibition zones for *Klebsiella pneumoniae, Pseudomonas aeruginosa*, and *Escherichia coli* were 11.67 ± 0.17 mm, 13.00 ± 0.58 mm, and 13.17 ± 1.17 mm, respectively. *Pseudomonas aeruginosa* was the most susceptible to chitosan at all three concentrations. The least susceptibility for chitosan was observed in *Klebsiella pneumoniae* at all three concentrations. The composition disparity between Gram-positive and Gram-negative cell walls results in varied interactions with chitosan. The structures of the cell walls of Gram-negative and Gram-positive bacteria are depicted in Figure 6. The protonated amino groups of chitosan interact with the anionic surface of Gram-negative bacterial membranes, composed of lipopolysaccharides, lipoproteins, and phospholipids. This interaction involves electrostatic binding but is most effective at lower concentrations. Furthermore, chitosan diffusion from agar wells is efficient at lower concentrations, explaining the best outcomes observed at 10 mg/mL for three Gram-negative bacteria: *Pseudomonas aeruginosa, Escherichia coli,* and *Klebsiella pneumoniae*. In the case of Gram-positive bacteria, whose cell walls consist of peptidoglycans without an outer membrane, chitosan directly obstructs the bacterial cell walls. This obstruction prevents the entry of nutrients and oxygen into the intracellular space, contributing to the antibacterial effect. The hydrophilicity discrepancy between Gram-negative and Gram-positive bacteria, owing to the presence of an outer membrane in Gram-negative bacteria, makes it more susceptible to the action of chitosan. This distinction may explain why *Staphylococcus aureus* did not yield detectable results in response to chitosan [64].

When compared separately with neem leaf extract and chitosan, the neem–chitosan composite demonstrated synergistic antibacterial activity with an overall enhanced effect. However, this was not observed in 20 mg/mL concentration against *Klebsiella pneumoniae*, 10 mg/mL concentration against *Pseudomonas aeruginosa*, and 5 mg/mL concentration against *Escherichia coli*. The combined effect against *Staphylococcus aureus* was not observed and chitosan showed no results. The highest susceptibility for the composite was observed in *Pseudomonas aeruginosa,* except at 20 mg/mL, at which the highest susceptibility was evident in *Staphylococcus aureus*. The lowest susceptibility for the composite was observed in *Klebsiella pneumoniae* at all three concentrations. The synergistic effect of the composite is due to the combined activity of neem leaf bioactive compounds and the action of chitosan against bacteria, as explained in the above sections.

Overall, it can be deduced that the combined effect of neem leaf extract and chitosan increased the antibacterial activity in the neem–chitosan composite. When considering all three samples, the sensitivity could be ranked from the highest to the lowest as *Pseudomonas aeruginosa > Staphylococcus aureus > Escherichia coli > Klebsiella pneumoniae*. Consequently, it can be concluded that the composite exhibits enhanced antibacterial activity against Gram-positive and Gram-negative bacteria.

### 3.7. Determination of the MIC and MBC

The results of minimum inhibitory concentration (MIC) and minimum bactericidal concentration (MBC) for neem leaf extract, chitosan, and neem–chitosan composite are shown in Table 5.

The MBC/MIC ratios were calculated to determine whether the materials tested were bacteriostatic or bactericidal on the bacterial species tested. MBC/MIC ratios greater than four were chosen as bacteriostatic, whereas ratios lesser than or equal to 4 were defined as bactericidal [65]. Neem extract demonstrated a bactericidal nature against all four strains. Chitosan against *Staphylococcus aureus* and neem–chitosan composite against *Escherichia coli* demonstrated a bactericidal nature, whereas, in all other instances, chitosan and neem–chitosan composite revealed a bacteriostatic nature. The content of bioactive compounds and the properties of chitosan, such as the degree of deacetylation, along with the type of bacteria (Gram-positive/Gram-negative), have been identified as influencing factors that yield different results in various scenarios. The mechanisms elaborated upon earlier under antibacterial activity can account for the observed bactericidal or bacteriostatic nature [64].

### 3.8. Drug Release Kinetics Study for Neem–Chitosan Composite

Various organs, like the stomach, intestine, etc., and body fluids, like saliva, blood, etc., have different pH and ionic strengths. The study was critical in revealing the controlled release behavior of the composite in various physiological conditions of the human body.

In general, in polymeric drug delivery systems, bioactive compounds are encapsulated in colloidal polymer particles through chemical crosslinking, ionic crosslinking, and ionic complexation [66]. Drug release from this polymeric system can occur through erosion, swelling, and diffusion. Diffusion releases the drug when a concentration gradient exists. The polymer chains make the barrier for diffusion and allow for sustained drug release. Swelling and erosion can also be associated with the process of diffusion. Fick’s Law of Diffusion mathematically explains diffusion. When the polymer system interacts with the surrounding media and gets broken down, swelling can occur, which releases the drug to the outside. Swelling occurs through the non-Fickian diffusion process, and the bioactive substances are delivered by erosion and diffusion. Erosion occurs when the polymer starts degrading from the edges and the drug is released. This involves swelling, diffusion, and dissolution [67,68].

The nature of the chitosan affects the release of neem bioactive compounds into the medium [69]. It depends on the interactions between the polymeric chains of chitosan. Here, the cumulative drug release showed a biphasic nature. An initial burst release occurs during the first few hours, and a steady release occurs during the next few hours [70].

**Figure 7:**
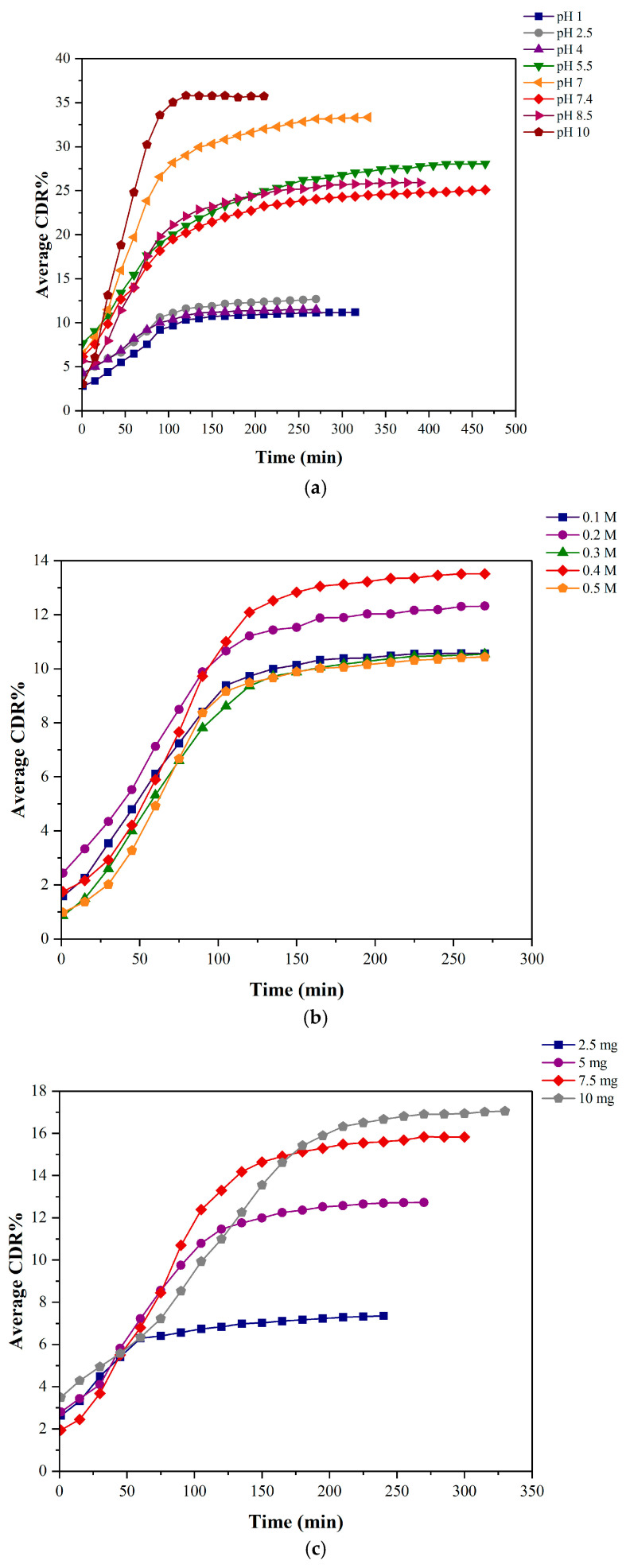
CDR% data of the drug composite under varying (**a**) pH, (**b**) ionic strength, and (**c**) drug weight.

Figure 7a–c depict the average cumulative drug release percentages of the neem–chitosan composite under varying pH, ionic strengths, and drug weights, respectively.

At pH levels 1, 2.5, and 4, the drug release after 3 h was approximately 11%, 12%, and 11%, respectively. Similarly, at pH 5.5, the release was approximately 27.5% after 6 h, at pH 7, it was 32.5% after 4 h, at pH 7.4, it was 24% after 5 h, at pH 8.5, it was 25% after 4 h, and at pH 10, it was 36% after 2 h. The release was higher in alkaline conditions than in acidic conditions. In NaCl concentrations of 0.1 M, 0.2 M, 0.3 M, 0.4 M, and 0.5 M, the drug release after 3 h was approximately 10%, 12%, 10%, 13%, and 10%, respectively. The release was approximately the same in all scenarios. The drug release reached a steady state after this stage. pH 7.4 was chosen as the most appropriate medium due to its sustained release properties and compatibility with intravenous drug administration, aligning with the physiological pH of the blood. The 0.4 M ionic medium was chosen as the most suitable medium due to its comparatively sustained and increased drug release. Accordingly, pH 7.4 and 0.4 M NaCl media were employed. For the combined medium, the drug release after 3 h was approximately 7%, 12%, and 15% for samples containing 2.5 mg, 5 mg, and 7.5 mg of the composite, respectively. Meanwhile, for the 10 mg sample, the release was 16.5% after 4 h. The drug release reached a steady state after this stage. The release was dose-dependent, whereas higher doses demonstrated a higher release. The 10 mg sample of the composite was chosen as the effective dose due to its sustained and increased release profile.

Six mathematical models were employed to analyze drug release kinetics, facilitating a better evaluation of drug release mechanisms. Table 6, Table 7 and Table 8 indicate the parameters of each model and the R^2^ values obtained for each curve.

The release of the drug in solutions of all the tested pH values and ionic strengths and the combined medium of pH 7.4 and 0.4 M NaCl used with varying drug dosages conforms well with the Korsmeyer–Peppas and Peppas–Sahlin models. Of all six models, these two had the highest R^2^, values ranging from 0.93 to 0.97. The R^2^ values of the Higuchi and Hixson–Crowell models ranged from 0.92 to 0.96 and from 0.89 to 0.93, respectively. The R^2^ value for both the Zero-order and First-order models ranged between 0.83 and 0.89.

In the Korsmeyer–Peppas model, *n* values (release exponent) less than or equal to 0.5 (*n* ≤ 0.5) indicate the dependence of the drug release mechanism on Fickian diffusion or quasi-Fickian diffusion [40]. However, the Fickian diffusion mechanism can be differentiated by *n* values < 0.43 for poly-dispersed systems. According to Ritger and Peppas, *n* values around 0.3 ± 0.01 are also possible for such systems [71]. Suggested here is that the drug is transported through polymer frameworks, not by solvent penetration [69]. In drug release through diffusion, water permeates the polymeric structure and transforms into a more flexible matrix, enabling the drug to diffuse [70]. The release constant (k) is proportional to the diffusion constant. Therefore, it depends on the neem and chitosan matrix’s physical and structural properties. The k value is an indication of the drug release rate. Hence, considering the k values, drug release percentages over time, and applicability, the best drug release profiles resulted in conditions including pH 7.4, 0.4 M NaCl, and 10 mg of drug composite dosage.

The Peppas–Sahlin model considers the Fickian and Case II relaxation contributions. The *m* value, the exponent coefficient, is related to the Fickian diffusion exponent. When the relaxational mechanism is insignificant, the value of *m* equals the *n* value of the KP Model. However, according to the above data (Table 6, Table 7 and Table 8), the *n* and *m* values are different, suggesting that neem release from chitosan depends on both Fickian diffusion and Case II relaxation. Furthermore, *n* values obtained for the KP model suggest that—at lower ionic strengths and lower pH values—neem release is mostly governed by Fickian diffusion.

Referring to the Higuchi model, although it does not apply to swellable drug release systems, it has been employed to study swellable systems due to its simplicity. For diffusion-controlled systems, the proportionality of CDR and the square root of time can be a good indication. However, this model was not employed for the drug release data in the current study, as further mathematical analysis is required. The Zero-order, First-order, and Hixson–Crowell models were also not utilized to analyze the drug release data of the current study. The R^2^ values obtained for all three models were significantly low compared to the first three models. The Hixson–Crowell model demonstrates the drug release based on dissolving velocity, which is proven not to be utilized by the neem–chitosan delivery system. Also, the zero-order model demonstrates the drug release based only on Case II relaxation, which is unsuitable for the current study. Hence, it can be inferred that the Korsmeyer–Peppas and Peppas–Sahlin models properly describe the drug release behavior of the neem–chitosan composite. Concurrently, the release of neem from the composite demonstrates a blend of Fickian diffusion and Case II relaxation, elucidated by the above-mentioned models.

Table 9 and Table 10 display the variations in cumulative drug release among the selected pH and media with different ionic strengths compared to the rest. A one-way analysis of variance (ANOVA) was executed to determine the presence of a statistically significant difference between the release profiles. If the *p*-value was less than 0.05, it was concluded that an important difference existed. If the *p*-value was more extensive than 0.05, it was concluded that a significant difference did not exist.

Table 9 shows a significant difference between release profiles of pH 1, 2.5, 4, 7, 7.4 and 10. The CDR of pH 5.5 and 8.5 did not indicate a significant difference. However, pH 7.4 was chosen due to its elevated release profile and suitability for intravenous drug administration. Table 10 confirms that a substantial difference is not evident in the four ionic media compared to the 0.4 M NaCl medium. Nevertheless, the 0.4 M ionic medium was chosen because it exhibited the highest cumulative drug release compared to the other media. These data can also be verified by Figure 7a,b, which show the CDR% of the drug composite under varying pH and ionic strengths.

## 4. Conclusions

The neem–chitosan composite was synthesized to serve as an effective drug endowed with antioxidant properties, potential to inhibit protein denaturation, and antibacterial properties, operating as a controlled/sustained drug delivery system. This was achieved by integrating ethanolic *Azadirachta indica* leaf extract with chitosan biopolymer, both of which were derived from natural sources. The neem leaf extract, rich in bioactive compounds such as phenols, tannins, flavonoids, glycosides, steroids, and terpenoids, contributed to its enhanced medicinal properties. Meanwhile, chitosan served as an exceptional drug carrier. Characterization of the composite through scanning electron microscopy (SEM) revealed the crystallization of neem extract on the porous structure of chitosan. Additionally, X-ray diffractometry (XRD) unveiled its crystallographic orientation. The novel drug exhibited superior antioxidant activity ranging from 3.56 ± 1.89% to 51.28 ± 1.14% for concentrations of 10 μg/mL to 70 μg/mL. Furthermore, it demonstrated the ability to inhibit the denaturation of egg albumin in the range of 59.68 ± 0.93% to 187.63 ± 3.53% for concentrations from 25 μg/mL to 1600 μg/mL.

The antibacterial effect of the composite resulted from a synergistic interaction between the neem leaf extract and chitosan, displaying enhanced activity against Gram-positive and Gram-negative bacteria through both bacteriostatic and bactericidal mechanisms. The drug release profiles obtained from different pH and ionic strength values meant that the drug release from the neem–chitosan composite involved a combination of Fickian diffusion and Case II relaxation, aligning more closely with the Korsemeyer–Peppas and Peppas–Sahlin models. This study primarily focused on an *in vitro* exploration of the medicinal properties of the novel drug, coupled with an analysis of its drug release profile. In future research on this topic, studies can be extended to *in vivo* investigations, enhancing the drug’s applicability for potential clinical use.

## Figures and Tables

**Table 1:** Phytochemicals present in the ethanolic extract of *Azadirachta indica* leaf.

Phytochemicals	Test	Present (+)/Absent (−)
Proteins	Ninhydrin test	−
Carbohydrates	Fehling’s test	−
	Benedict’s test	−
	Iodine test	−
Phenols and Tannins	-	+
Flavonoids	Alkaline reagent test	+
Saponins	Foam test	−
Glycosides	Salkowski’s test	+
	Keller-Gilani test	−
Steroids	-	+
Terpenoids	-	+
Alkaloids	-	−

**Table 2:** Crystallographic parameters were calculated from XRD data.

**Sample**	**2θ°**	**L (nm)**	**d (nm)**	**L/d**
Neem	24.44	12.7275	0.3638	34.99
Chitosan	19.36	54.7894	0.4579	119.64
Composite	19.48	60.3622	0.4551	132.62

**Table 3:** Related studies conducted to test the antioxidant activity using DPPH assay.

Composite	Highest %RSA	Concentration	Reference
Chitosan–Mango leaf extract film	87.16%	5%	[51]
Curcumin–chitosan polymer	53.2%	1%	[52]
Black rice extract-loaded chitosan and polyvinyl alcohol composite	55.35 ± 0.54%	30%	[53]
Chitosan films incorporated with green tea extract	~50%	20%	[54]
Copper oxide-incorporated chitosan–neem seed biocomposites	59%	100 μg/mL	[55]
Grape extract loaded in chitosan nanoparticles	62.8%	1000 mg/mL	[55]
Neem–chitosan composite	51.28 ± 1.14%	70 μg/mL	Present study

**Table 4:** Related studies conducted to test the inhibition of denaturation of egg albumin.

Composite	Highest %Inhibition	Concentration	Reference
Magnesium Oxide-Doped Chitosan/Polyvinyl Alcohol With *Catharanthus roseus*	~90%	100 μg/mL	[56]
Chitosan Thiocolchicoside-Lauric Acid Nanogel	80%	50 μg/mL	[57]
Chitosan/oxidized pectin/PVA blend film	79.07%	750 μg/mL	[58]
Curcumin-loaded chitosan nanoparticles	66%	600 μg/mL	[59]
Chitosan nanoparticles of combination of gallic acid and rutin	76.70 ± 0.27%	500 μg/mL	[60]
*C. arvensis* alginate/chitosan NP extracts	62.38 ± 2.14%	1000 μg/mL	[61]
Neem–chitosan composite	187.63 ± 3.53%	1600 μg/mL	Present study

**Table 5:** MIC and MBC results of neem, chitosan and composite.

Bacterial Strains	MIC (mg/mL)			MBC (mg/mL)			MBC/MIC		
	Neem:	Chi:	Com:	Neem:	Chi:	Com:	Neem:	Chi:	Com:
*Klebsiella pneumoniae*	1.25	1.25	1.25	2.5	10	20	2	8	16
*Pseudomonas aeruginosa*	1.25	1.25	1.25	5	10	20	4	8	16
*Escherichia coli*	1.25	1.25	1.25	5	10	5	4	8	4
*Staphylococcus aureus*	1.25	1.25	1.25	5	5	40	4	4	32

**Table 6:** Model parameters and R^2^ values for the drug release in media with different pH values.

	pH 1	pH 2.5	pH 4	pH 5.5	pH 7	pH 7.4	pH 8.5	pH 10
Korsmeyer–Peppas Model								
K	1.9999	2.6005	3.0885	5.2887	5.3988	5.3260	4.2837	3.9934
*n*	0.3145	0.2929	0.2457	0.2811	0.3285	0.2632	0.3171	0.4337
R^2^	0.9497	0.9609	0.9562	0.9790	0.9578	0.9644	0.9464	0.9454
Peppas–Sahlin Model								
K_d_	0.9878	1.7337	1.8652	2.2883	2.3026	2.3012	1.9105	1.8202
K_r_	1.1626	1.0627	1.3684	3.3251	3.4856	3.3185	2.7034	2.6061
*m*	0.1819	0.1861	0.1555	0.1598	0.1845	0.1505	0.1790	0.2404
R^2^	0.9489	0.9615	0.9566	0.9787	0.9567	0.9633	0.9451	0.9438
Higuchi Model								
K_H_	0.7547	0.9051	0.8465	1.5424	2.1751	1.4047	1.5766	2.8909
R^2^	0.9360	0.9584	0.9471	0.9648	0.9414	0.9353	0.9268	0.9396
Zero-Order Model								
K_0_	0.0492	0.0637	0.0592	0.0829	0.1388	0.0752	0.0925	0.2323
R^2^	0.8548	0.8965	0.8710	0.8903	0.8567	0.8405	0.8368	0.8620
First-Order Model								
K_1_	0.1112	0.1441	0.1338	0.1895	0.3176	0.1716	0.2113	0.5319
R^2^	0.8548	0.8965	0.8710	0.8903	0.8567	0.8405	0.8368	0.8620
Hixson–Crowell model								
K_HC_	0.0328	0.0424	0.0394	0.0553	0.0925	0.0501	0.0617	0.1549
R^2^	0.9110	0.9348	0.9150	0.9292	0.9134	0.8962	0.9004	0.9255

**Table 7:** Model parameters and R^2^ values for the drug release in media with different NaCl concentrations.

	0.1 M	0.2 M	0.3 M	0.4 M	0.5 M
Korsmeyer–Peppas Model					
K	1.2540	1.6191	0.8161	0.8909	0.7715
*n*	0.3999	0.3782	0.4773	0.5075	0.4870
R^2^	0.9572	0.9605	0.9605	0.9513	0.9416
Peppas–Sahlin Model					
K_d_	0.6211	0.7437	0.4752	0.5271	0.3457
K_r_	0.6717	0.8299	0.4931	0.5426	0.4809
*m*	0.2369	0.2294	0.2662	0.2818	0.2735
R^2^	0.9548	0.9589	0.9591	0.9501	0.9392
Higuchi Model					
K_H_	0.7515	0.8689	0.7266	0.9256	0.7219
R^2^	0.9504	0.9549	0.9592	0.9515	0.9408
Zero-Order Model					
K_0_	0.0533	0.0616	0.0520	0.0665	0.0517
R^2^	0.8788	0.8892	0.8973	0.9029	0.8785
First-Order Model					
K_1_	0.1204	0.1394	0.1173	0.1507	0.1166
R^2^	0.8788	0.8892	0.8973	0.9029	0.8785
Hixson–Crowell model					
K_HC_	0.0356	0.0411	0.0347	0.0443	0.0345
R^2^	0.9313	0.9357	0.9452	0.9489	0.9362

**Table 8:** Model parameters and R^2^ values for the drug release in the combined medium varying the dosage of the drug composite.

	2.5 mg	5 mg	7.5 mg	10 mg
Korsmeyer–Peppas Model				
K	2.3891	1.5709	1.1886	0.9716
*n*	0.2133	0.3901	0.4742	0.5107
R^2^	0.9674	0.9613	0.9640	0.9694
Peppas–Sahlin Model				
K_d_	1.2253	0.7562	0.6355	0.5730
K_r_	1.2325	0.8455	0.6989	0.5790
*m*	0.1312	0.2310	0.2680	0.2853
R^2^	0.9668	0.9605	0.9552	0.9695
Higuchi Model				
K_H_	0.5742	0.8959	1.0384	1.0283
R^2^	0.9383	0.9585	0.9638	0.9690
Zero-Order Model				
K_0_	0.0422	0.0636	0.0706	0.0672
R^2^	0.8434	0.9005	0.9201	0.9489
First-Order Model				
K_1_	0.0945	0.1440	0.1604	0.1527
R^2^	0.8434	0.9005	0.9201	0.9489
Hixson–Crowell model				
K_HC_	0.0282	0.0424	0.0471	0.0448
R^2^	0.8935	0.9425	0.9598	0.9722

**Table 9:** ANOVA results: average %CDR of varying pH values compared with the %CDR of pH 7.4.

pH	1	2.5	4	5.5	7	8.5	10
*p*-value	<0.001	<0.001	<0.001	0.1674	0.0026	0.8601	0.0102
F-value	90.22	65.16	75.18	1.95	9.98	0.03	7.18

**Table 10:** ANOVA results: average %CDR of varying NaCl concentrations compared with the %CDR of the media having 0.4 M NaCl ionic strength.

NaCl Conc.	0.1 M	0.2 M	0.3 M	0.5 M
*p*-value	0.1736	0.7859	0.1018	0.0946
F-value	1.93	0.07	2.82	2.95

## Data Availability

Data are contained within the article.
